# Cramer-Rao Lower Bound Evaluation for Linear Frequency Modulation Based Active Radar Networks Operating in a Rice Fading Environment

**DOI:** 10.3390/s16122072

**Published:** 2016-12-06

**Authors:** Chenguang Shi, Sana Salous, Fei Wang, Jianjiang Zhou

**Affiliations:** 1 Key Laboratory of Radar Imaging and Microwave Photonics, Ministry of Education, Nanjing University of Aeronautics and Astronautics, Nanjing 210016, China; scg_space@163.com (C.S.); wangxiaoxian@nuaa.edu.cn (F.W.); 2 School of Engineering and Computing Sciences, Durham University, Durham DH1 3DE, UK; salous.durham@gmail.com

**Keywords:** Cramer-Rao lower bound (CRLB), Fisher information matrix (FIM), joint parameter estimation, linear frequency modulation (LFM) signal, Rician target, active radar networks

## Abstract

This paper investigates the joint target parameter (delay and Doppler) estimation performance of linear frequency modulation (LFM)-based radar networks in a Rice fading environment. The active radar networks are composed of multiple radar transmitters and multichannel receivers placed on moving platforms. First, the log-likelihood function of the received signal for a Rician target is derived, where the received signal scattered off the target comprises of dominant scatterer (DS) component and weak isotropic scatterers (WIS) components. Then, the analytically closed-form expressions of the Cramer-Rao lower bounds (CRLBs) on the Cartesian coordinates of target position and velocity are calculated, which can be adopted as a performance metric to access the target parameter estimation accuracy for LFM-based radar network systems in a Rice fading environment. It is found that the cumulative Fisher information matrix (FIM) is a linear combination of both DS component and WIS components, and it also demonstrates that the joint CRLB is a function of signal-to-noise ratio (SNR), target’s radar cross section (RCS) and transmitted waveform parameters, as well as the relative geometry between the target and the radar network architectures. Finally, numerical results are provided to indicate that the joint target parameter estimation performance of active radar networks can be significantly improved with the exploitation of DS component.

## 1. Introduction

### 1.1. Related Works and Motivation

With widely separated transmitters and receivers, the distributed radar networks, also known as spatial distributed multiple-input multiple-output (MIMO) radar systems [1,2,3], can view the target from different aspect angles and provide spatial and signal diversities. To be specific, for a distributed radar network system with *M* transmitters and *N* receivers, the various transmitter-receiver pairs observe the different aspects of the target. In this way, we can obtain the equivalent of MN radars by optimizing the selection of the transmitted signals from different transmitters. However, the conventional radar observes only single aspect of the target. As we can conclude in [4], the advantage of the radar network is that the average received energy across all the transmitter-receiver pairs is approximately constant, and it overcomes deep fades other than the conventional systems. Therefore, the radar network systems have attracted considerable attention and on a path from theory to practice [4,5,6,7,8,9,10].

The Cramer-Rao lower bound (CRLB) is an important tool for analyzing the performance of radar networks, which can provide the smallest variance estimates for any unbiased estimation [11,12]. The mean-square error (MSE) of the maximum likelihood estimator (MLE) is close to the CRLB when the high signal-to-noise ratio (SNR) is satisfied. It is also worth mentioning that the performance of multiple signal classification (MUSIC) in computational time-reversal (TR) applications is studied in [13,14], where the closed-form MSE matrix of TR-MUSIC is calculated for the single-frequency case in multistatic co-located and non co-located scenarios. Simulation results show that TR-MUSIC can predict a more accurate MSE than CRLB, while it is a sub-optimal estimator since it does not asymptotically achieve the CRLB as the MLE. In the last couple of years, there is a growing interest on the CRLB studies for the target estimation performance of distributed radar networks [11,12,13,14,15,16,17,18,19]. The authors in [11] derive the analytical expressions of CRLB for both noncoherent mode and coherent mode in MIMO radar systems, which shows that the CRLB is inversely proportional to the carrier frequency and signals averaged effective bandwidth. In [12], the problem of target parameter estimation for noncoherent MIMO radar is addressed, and the joint CRLB of target position and velocity is computed. Reference [15] further extends the results in [12] to a multiple targets scenario. Later, He et al. investigate the coherent MIMO radar performance when the oscillators at each transmitter and receiver are aligned in phase [16]. The work in [17] studies the target localization accuracy for MIMO radar systems with static phase errors. In [18], the CRLBs of the joint time delay and Doppler shift estimation are derived for an extended target, and the effects of transmitted waveform parameters on the CRLBs are analyzed. Assuming that the approximation state of the target is unknown without previous target detection, a generalized CRLB for distributed active and passive radar networks is calculated in [19].

Recently, the CRLB has been investigated and applied to passive radar systems that employ signals of opportunity as illuminators for target detection, estimation and tracking [20,21,22,23,24]. Since passive radar does not use its own transmitter to radiate electromagnetic wave, it has been a potential technology for low cost, low probability of intercept (LPI) [25,26,27], antijamming and other advantages. The authors in [20] present the CRLB analysis for the joint target estimation of position and velocity in a frequency modulation (FM) based passive radar networks. In [21,22,23,24], the modified CRLB (MCRLB) is employed as a good alternative to the classical CRLB due to the presence of random parameters in the transmitted waveforms, which has been shown to offer a looser bound in practical applications. The target estimation performance of a universal mobile telecommunications systems (UMTS)-based passive multistatic radar and an orthogonal frequency-division multiplexing (OFDM)-based passive radar network in a line-of-sight (LoS) environment is analyzed in [23,24] respectively, where the Rician target model is composed of two components, that is, fixed amplitude or dominant scatterer (DS) and weak isotropic scatterers (WIS). It is shown that the target estimation accuracy will be increased with an increase in reflection coefficient, number of transmitter-receiver pairs, the choice of the transmitter-receiver pairs and duration time. Furthermore, the work in [28] proposes two transmitter of opportunity selection algorithms for FM-based passive radar network systems, which are formulated as knapsack problems (KPs) and tackled with greedy selection approaches. On the basis of the research mentioned above, almost all of previous works focus on stationary platforms. The CRLB analysis for joint moving target position and velocity estimation of linear frequency modulation (LFM) based active radar networks with sensors placed on moving platforms operating in a Rice fading environment, which has not been considered, needs to be investigated.

### 1.2. Major Contributions

The major contributions of this paper are fourfold:

(1) We formulate the linear frequency modulation (LFM) signal model and derive the log-likelihood function of the received signal for a Rician target. The Rician target model is composed of DS component and WIS components, which are the signals received after striking from the target [21,22]. It is worth pointing out here that [15,16,27] only study the target parameter estimation accuracy limits either when the target’ radar cross section (RCS) observes as a Rayleigh model in a non-coherent scenario or the target is modeled as a point target in a coherent scenario for all the transmitter-receiver pairs [23]. While utilizing a Rician target model, the estimation performance can be generalized and evaluated when the target has different RCS models for different transmitter-receiver pairs.
(2) On the basis of the previous works [15,16,17,18,19,20,21,22,23,24,29], almost all the studies concentrate on stationary platforms. In this paper, we build an LFM-based active radar network configuration and extend it to a more general case, which consist of multiple radar transmitters and multichannel receivers placed on moving platforms. On the other hand, only the CRLBs for LFM-based bistatic radar channels are computed in [29]. To the best of our knowledge, the CRLB for an LFM-based radar network has not been derived. Thus, the joint CRLB for position and velocity estimation of a Rician target in LFM-based radar networks is computed, where we assume that the signals scattered off the target due to different radar transmitters can be received and separated at the receivers. The cumulative Fisher information matrix (FIM) can be factored into two terms: one term accounting for the effect of the DS component, and another incorporating the effect of the WIS components.
(3) Simulation results have shown that the DS component can be exploited to decrease the target parameter estimation errors, which is due to the fact that the reception of DS component increases the received SNR at the radar receiver. Previous results in [20,29] only show that the CRLB is a function of the signal parameters as well as the geometry between the target and the radar network architecture. In this paper, the effects of SNR and target’s RCS on the target parameter estimation performance are also analyzed. It is demonstrated that the joint CRLB is not only a function of SNR, target’s RCS and transmitted waveform parameters, but also a function of the geometry between the target and the active radar network systems.
(4) The closed-form expressions of CRLB can be used as a performance metric to access the target estimation performance for LFM-based active radar networks in a Rice fading environment. Since the DS component can be exploited to increase the received SNR at the receiver, the geometry-dependent CRLB analysis will open up a new dimension for active radar network systems by aiding the optimal power allocation for radar networks to achieve a given estimation requirement with the minimum system cost.

### 1.3. Outline of the Paper

The rest of the paper is organized as follows. Section 1 describes the signal model for LFM-based radar networks. In Section 2, the joint CRLB is computed for target position and velocity estimation by deriving the closed-form expressions of FIM. The numerical simulations are provided to demonstrate our analytical results in Section 3. Finally, conclusion remarks are drawn with potential future work in Section 4.

**Notation:** The superscript *T* represents the transpose operator; E{·} and ${\left(\cdot \right)}^{*}$ represent the expectation and conjugation operators, respectively. |·| denotes the absolute value, ℜ{·} is the real part, and ℑ{·} is the imaginary part. $S_{i}\left(f\right)$ denotes the Fourier transform of $s_{i}\left(t\right)$ .

## 2. Signal Model

Consider a active radar network architecture comprising of $N_{T}$ radar transmitters and $N_{R}$ multichannel receivers. Let the *i*th radar transmitter and the *j*th receiver be located at $\vec{p_{i}^{t}}=\left[x_{i}^{t},y_{i}^{t}\right]$ and $\vec{p_{j}^{r}}=\left[x_{j}^{r},y_{j}^{r}\right]$ respectively, in a 2-dimensional Cartesian coordinate system for simplicity. The target position and velocity are supposed to be deterministic unknown and denoted by $\vec{p}=\left[x,y\right]$ and $\vec{v}=\left[v_{x},v_{y}\right]$ . We define the unknown target state vector:

(1) $$U={\left[x,y,v_{x},v_{y}\right]}^{T}.$$

Without loss of generality, we will concentrate on a single target scenario. However, the results can be extended to multiple targets.

Let $\tau_{ij}$ represent the bistatic time delays corresponding to the path between the *i*th radar transmitter, moving target, and the *j*th radar receiver, which is a function of the unknown target position $\vec{p}=\left[x,y\right]$ :

(2) $$\begin{array}{cc} \tau_{ij} & =\frac{\sqrt{{\left(x-x_{i}^{t}\right)}^{2}+{\left(y-y_{i}^{t}\right)}^{2}}+\sqrt{{\left(x-x_{j}^{r}\right)}^{2}+{\left(y-y_{j}^{r}\right)}^{2}}}{c}\;\\ & =\frac{∥\vec{p}-\vec{p_{i}^{t}}∥+∥\vec{p}-\vec{p_{j}^{r}}∥}{c}, \end{array}$$

where *c* is the speed of light, $∥\vec{p}-\vec{p_{i}^{t}}∥$ denotes the distance from the *i*th radar transmitter to the target and $∥\vec{p}-\vec{p_{j}^{r}}∥$ denotes the distance from the target to the *j*th receiver, respectively. In this paper, the *i*th radar transmitter and the *j*th multichannel receiver are moving with velocities $\vec{v_{i}^{t}}=\left[v_{x,i}^{t},v_{y,i}^{t}\right]$ and $\vec{v_{j}^{r}}=\left[v_{x,j}^{r},v_{y,j}^{r}\right]$ , respectively. With the aforementioned positions/velocities of the target, the radar transmitters and receivers, the Doppler shift of the moving target corresponding to the ij th path is the time rate of change of the total ij th path length:

(3) $$f_{D_{ij}}=\frac{1}{\lambda }\left[\frac{\partial ∥\vec{p}-\vec{p_{i}^{t}}∥}{\partial t}+\frac{\partial ∥\vec{p}-\vec{p_{j}^{r}}∥}{\partial t}\right],$$

where *λ* denotes the carrier wavelength, $\frac{\partial ∥\vec{p}-\vec{p_{i}^{t}}∥}{\partial t}$ and $\frac{\partial ∥\vec{p}-\vec{p_{j}^{r}}∥}{\partial t}$ are the relative velocities for the *i*th radar transmitter and the *j*th receiver, respectively. Then, we have:

(4) $$\begin{array}{cc} f_{D_{ij}} & =\frac{1}{\lambda }\left[v_{x}\left(\frac{x-x_{i}^{t}}{∥\vec{p}-\vec{p_{i}^{t}}∥}+\frac{x-x_{j}^{r}}{∥\vec{p}-\vec{p_{j}^{r}}∥}\right)\right]+\frac{1}{\lambda }\left[v_{y}\left(\frac{y-y_{i}^{t}}{∥\vec{p}-\vec{p_{i}^{t}}∥}+\frac{y-y_{j}^{r}}{∥\vec{p}-\vec{p_{j}^{r}}∥}\right)\right]\;\\ & +\frac{1}{\lambda }\left[v_{x,i}^{t}\frac{x-x_{i}^{t}}{∥\vec{p}-\vec{p_{i}^{t}}∥}+v_{y,i}^{t}\frac{y-y_{i}^{t}}{∥\vec{p}-\vec{p_{i}^{t}}∥}\right]+\frac{1}{\lambda }\left[v_{x,j}^{r}\frac{x-x_{j}^{r}}{∥\vec{p}-\vec{p_{j}^{r}}∥}+v_{y,j}^{r}\frac{y-y_{j}^{r}}{∥\vec{p}-\vec{p_{j}^{r}}∥}\right], \end{array}$$

which is a function of the unknown target position $\vec{p}=\left[x,y\right]$ and velocity $\vec{v}=\left[v_{x},v_{y}\right]$ .

The LFM signal transmitted by the *i*th radar transmitter is given by [29]:

(5) $$s_{i}\left(t\right)=\frac{1}{\sqrt{N}}\sum_{n=0}^{N-1}u_{i}\left(t-nT_{R}\right),$$

where

ui(t)={(6a)1Tejπkt2,|t|≤T2,(6b)0,elsewhere

*N* is the number of subpulses for each transmitted burst, $T_{R}$ is the pulse repetition interval (PRI) and *T* is the duration of each pulse, such that $T<T_{R}/2$ . Moreover, $kT^{2}=BT$ represents the effective time-bandwidth product of the signal and *B* denotes the total frequency derivation. Note that each transmitter-receiver pair has its own angle of view for the target because of the widely spaced antennas that leads to different attenuation factors [20]. It is assumed that the signals from different radar transmitters are supposed to be received and processed at the multichannel radar receivers. For a Rician target, it consists of a DS and many independent WIS. In this paper, by utilizing the Rician target model [23], the reflection coefficient $\zeta_{ij}$ is modeled as a complex Gaussian random variable with mean $d_{ij}$ and variance $\sigma^{2}$ , i.e., $\zeta_{ij}∼\mathrm{CN}\left(d_{ij},\sigma^{2}\right)$ . Then, the signal from the *i*th radar transmitter arriving at the *j*th receiver can be expressed as:

(7) $$r_{ij}\left(t\right)=\zeta_{ij}s_{i}\left(t-\tau_{ij}\right)e^{j2\pi f_{D_{ij}}\left(t-\tau_{ij}\right)}+w_{ij}\left(t\right),$$

where $w_{ij}\left(t\right)$ denotes the additive zero-mean white Gaussian noise of variance corresponding to the ij th path, i.e., $w_{ij}∼\mathrm{CN}\left(0,\sigma_{n}^{2}\right)$ , which is independent to $\zeta_{ij}$ . We assume that the parameters $d_{ij}$ , $\sigma^{2}$ and $\sigma_{n}^{2}$ are deterministic and known.

Following the concepts and derivations in [12,23], the likelihood ratio of the ij th transmitter-receiver pair can be given by:

(8) $$\begin{array}{cc} \Lambda (r_{ij}\left(t\right);U) & =\exp\{\frac{\sigma^{2}}{\sigma^{2}+\sigma_{n}^{2}}{\left|\int_{-\infty }^{+\infty }r_{ij}\left(t\right)s_{i}^{*}\left(t-\tau_{ij}\right)e^{-j2\pi f_{D_{ij}}\left(t-\tau_{ij}\right)}dt\right|}^{2}\;\\ & -\frac{1}{\sigma^{2}+\sigma_{n}^{2}}{\left|\int_{-\infty }^{+\infty }a_{r_{ij}}s_{i}^{*}\left(t-\tau_{ij}\right)e^{-j2\pi f_{D_{ij}}\left(t-\tau_{ij}\right)}dt\right|}^{2}\;\\ & +\frac{2}{\sigma^{2}+\sigma_{n}^{2}}ℜ\left[\int_{-\infty }^{+\infty }r_{ij}\left(t\right)s_{i}^{*}\left(t-\tau_{ij}\right)e^{-j2\pi f_{D_{ij}}\left(t-\tau_{ij}\right)}dt\right.\;\\ & \left.\times d_{r_{ij}}^{*}s_{i}\left(t-\tau_{ij}\right)e^{j2\pi f_{D_{ij}}\left(t-\tau_{ij}\right)}dt\right]\}+\left(\frac{\sigma_{n}^{2}}{\sigma^{2}+\sigma_{n}^{2}}\right), \end{array}$$

where $d_{r_{ij}}$ represents the mean of the received signal $r_{ij}\left(t\right)$ , i.e., $d_{r_{ij}}=d_{ij}s_{i}\left(t-\tau_{ij}\right)e^{j2\pi f_{D_{ij}}\left(t-\tau_{ij}\right)}$ . Furthermore, the log-likelihood ratio is written as:

(9) $$\begin{array}{cc} L(r_{ij}\left(t\right);U) & =\frac{\sigma^{2}}{\sigma^{2}+\sigma_{n}^{2}}{\left|\int_{-\infty }^{+\infty }r_{ij}\left(t\right)s_{i}^{*}\left(t-\tau_{ij}\right)e^{-j2\pi f_{D_{ij}}\left(t-\tau_{ij}\right)}dt\right|}^{2}\;\\ & -\frac{1}{\sigma^{2}+\sigma_{n}^{2}}{\left|\int_{-\infty }^{+\infty }d_{r_{ij}}s_{i}^{*}\left(t-\tau_{ij}\right)e^{-j2\pi f_{D_{ij}}\left(t-\tau_{ij}\right)}dt\right|}^{2}\;\\ & +\frac{2}{\sigma^{2}+\sigma_{n}^{2}}ℜ\left\{\int_{-\infty }^{+\infty }r_{ij}\left(t\right)s_{i}^{*}\left(t-\tau_{ij}\right)e^{-j2\pi f_{D_{ij}}\left(t-\tau_{ij}\right)}dt\right.\;\\ & \left.\times d_{r_{ij}}^{*}s_{i}\left(t-\tau_{ij}\right)e^{j2\pi f_{D_{ij}}\left(t-\tau_{ij}\right)}dt\right\}+\ln\left(\frac{\sigma_{n}^{2}}{\sigma^{2}+\sigma_{n}^{2}}\right). \end{array}$$

Due to the fact that the radar transmitters and receivers are widely separated, the received signals $r_{ij}\left(t\right)$ are mutually independent for different transmitter-receiver pairs. Therefore, the joint log-likelihood ratio across all the transmitter-receiver pairs can be written as the sum of all single transmitter-receiver pair log-likelihood ratios:

(10) $$\begin{array}{cc} L(r(t);U) & =\sum_{i=1}^{N_{T}}\sum_{j=1}^{N_{R}}L\left(r_{ij}\left(t\right);U\right)\;\\ & =\sum_{i=1}^{N_{T}}\sum_{j=1}^{N_{R}}\left(\Gamma_{ij}^{1}-\Gamma_{ij}^{2}+\Gamma_{ij}^{3}\right)+C, \end{array}$$

where $r(t)={\left[r_{11}\left(t\right),r_{12}\left(t\right),\cdots ,r_{N_{T}N_{T}}\left(t\right)\right]}^{T}$ is the observed signals from the entire set of the receivers. $\Gamma_{ij}^{1}$ , $\Gamma_{ij}^{2}$ and $\Gamma_{ij}^{3}$ denote the first, second and third terms in (9), respectively. The constant $C=\sum_{i=1}^{N_{T}}\sum_{j=1}^{N_{R}}\ln\left(\frac{\sigma_{n}^{2}}{\sigma^{2}+\sigma_{n}^{2}}\right)$ is independent of the target state vector U . Therefore, the MLE of the unknown target state vector U can be expressed as:

(11) $$\begin{array}{cc} {\hat{U}}_{\mathrm{ML}} & =\arg\max_{U}L\left(r(t);U\right)\;\\ & =\arg\max_{U}\sum_{i=1}^{N_{T}}\sum_{j=1}^{N_{R}}L\left(r_{ij}\left(t\right);U\right)\;\\ & =\arg\max_{U}\sum_{i=1}^{N_{T}}\sum_{j=1}^{N_{R}}\left(\Gamma_{ij}^{1}-\Gamma_{ij}^{2}+\Gamma_{ij}^{3}\right), \end{array}$$

where ${\hat{U}}_{\mathrm{ML}}$ represents the MLE of the unknown parameter vector U .

## 3. Derivation of Joint Cramer-Rao Lower Bound

It is discussed in [12,16] that the CRLB indicates the smallest variance estimate of any unbiased estimate, which can be adopted as a performance metric in parameter estimation problems because that the CRLB is close to the MSE of the MLE when the high SNR is satisfied. Using the derivations in [12,21], the FIM is a 4×4 matrix related to the second-order derivatives of the joint log-likelihood function:

(12) $$\begin{array}{cc} J(U) & =\left({▽}_{U}Q^{T}\right)J\left(Q\right){\left({▽}_{U}Q^{T}\right)}^{T}\;\\ & =\left({▽}_{U}Q^{T}\right)\left(-E_{r(t);U}\left\{{▽}_{Q}{\left[{▽}_{Q}L\left(r\left(t\right);U\right)\right]}^{T}\right\}\right){\left({▽}_{U}Q^{T}\right)}^{T}, \end{array}$$

where we define Q as an alternative representation of the unknown parameter vector:

(13) $$Q={\left[\tau_{ij},f_{D_{ij}}\right]}^{T}\left(\forall i,j\right).$$

We first derive the Jacobian matrix $\left({▽}_{U}Q^{T}\right)$ , whose entries can be obtained by taking the first-order derivatives of the time-delays in (2) and the Doppler shifts in (4) with respect to target positions:

(14) $$\frac{\partial \tau_{ij}}{\partial x}\equiv \frac{1}{c}\left(\frac{x-x_{i}^{t}}{∥\vec{p}-\vec{p_{i}^{t}}∥}+\frac{x-x_{j}^{r}}{∥\vec{p}-\vec{p_{j}^{r}}∥}\right),$$

(15) $$\frac{\partial \tau_{ij}}{\partial y}\equiv \frac{1}{c}\left(\frac{y-y_{i}^{t}}{∥\vec{p}-\vec{p_{i}^{t}}∥}+\frac{y-y_{j}^{r}}{∥\vec{p}-\vec{p_{j}^{r}}∥}\right),$$

(16) $$\begin{array}{cc} \frac{\partial f_{D_{ij}}}{\partial x} & \equiv \frac{1}{\lambda }\left\{v_{x}\left[\frac{{\left(y-y_{i}^{t}\right)}^{2}}{{∥\vec{p}-\vec{p_{i}^{t}}∥}^{3}}+\frac{{\left(y-y_{j}^{r}\right)}^{2}}{{∥\vec{p}-\vec{p_{j}^{r}}∥}^{3}}\right]+v_{y}\left[-\frac{\left(x-x_{i}^{t}\right)\left(y-y_{j}^{r}\right)}{{∥\vec{p}-\vec{p_{i}^{t}}∥}^{3}}-\frac{\left(x-x_{j}^{r}\right)\left(y-y_{j}^{r}\right)}{{∥\vec{p}-\vec{p_{j}^{r}}∥}^{3}}\right]\right.\;\\ & \left.+\left[v_{x,i}^{t}\frac{{\left(y-y_{i}^{t}\right)}^{2}}{∥\vec{p}-\vec{p_{i}^{t}}{∥}^{3}}-v_{y,i}^{t}\frac{\left(x-x_{i}^{t}\right)\left(y-y_{i}^{t}\right)}{∥\vec{p}-\vec{p_{i}^{t}}{∥}^{3}}\right]+\left[v_{x,j}^{r}\frac{{\left(y-y_{j}^{r}\right)}^{2}}{∥\vec{p}-\vec{p_{j}^{r}}{∥}^{3}}-v_{y,j}^{r}\frac{\left(x-x_{j}^{r}\right)\left(y-y_{j}^{r}\right)}{∥\vec{p}-\vec{p_{j}^{r}}{∥}^{3}}\right]\right\}, \end{array}$$

(17) $$\begin{array}{cc} \frac{\partial f_{D_{ij}}}{\partial y} & \equiv \frac{1}{\lambda }\left\{v_{x}\left[-\frac{\left(x-x_{i}^{t}\right)\left(y-y_{j}^{r}\right)}{{∥\vec{p}-\vec{p_{i}^{t}}∥}^{3}}-\frac{\left(x-x_{j}^{r}\right)\left(y-y_{j}^{r}\right)}{{∥\vec{p}-\vec{p_{j}^{r}}∥}^{3}}\right]\right.+v_{y}\left[\frac{{\left(x-x_{i}^{t}\right)}^{2}}{{∥\vec{p}-\vec{p_{i}^{t}}∥}^{3}}+\frac{{\left(x-x_{j}^{r}\right)}^{2}}{{∥\vec{p}-\vec{p_{j}^{r}}∥}^{3}}\right]\;\\ & \left.+\left[-v_{x,i}^{t}\frac{\left(x-x_{i}^{t}\right)\left(y-y_{i}^{t}\right)}{∥\vec{p}-\vec{p_{i}^{t}}{∥}^{3}}+v_{y,i}^{t}\frac{{\left(x-x_{i}^{t}\right)}^{2}}{∥\vec{p}-\vec{p_{i}^{t}}{∥}^{3}}\right]+\left[-v_{x,j}^{r}\frac{\left(x-x_{j}^{r}\right)\left(y-y_{j}^{r}\right)}{∥\vec{p}-\vec{p_{j}^{r}}{∥}^{3}}+v_{y,j}^{r}\frac{{\left(x-x_{j}^{r}\right)}^{2}}{∥\vec{p}-\vec{p_{j}^{r}}{∥}^{3}}\right]\right\}, \end{array}$$

Similarly, the derivatives with respect to the target velocities can be calculated as:

(18) $$\frac{\partial \tau_{ij}}{\partial v_{x}}\equiv 0,$$

(19) $$\frac{\partial \tau_{ij}}{\partial v_{y}}\equiv 0,$$

(20) $$\frac{\partial f_{D_{ij}}}{\partial v_{x}}\equiv \frac{1}{\lambda }\left(\frac{x-x_{i}^{t}}{∥\vec{p}-\vec{p_{i}^{t}}∥}+\frac{x-x_{j}^{r}}{∥\vec{p}-\vec{p_{j}^{r}}∥}\right),$$

(21) $$\frac{\partial f_{D_{ij}}}{\partial v_{y}}\equiv \frac{1}{\lambda }\left(\frac{y-y_{i}^{t}}{∥\vec{p}-\vec{p_{i}^{t}}∥}+\frac{y-y_{j}^{r}}{∥\vec{p}-\vec{p_{j}^{r}}∥}\right).$$

After lengthy algebraic derivations, the FIM J(Q) can be correspondingly expressed by:

(22) $$\begin{array}{cc} J(Q) & =-E_{r(t);U}\left\{\left[{▽}_{Q}L\left(r\left(t\right);U\right)\right]{\left[{▽}_{Q}L\left(r\left(t\right);U\right)\right]}^{T}\right\}\;\\ & =-E_{r(t);U}\left\{{▽}_{Q}{\left[{▽}_{Q}L\left(r\left(t\right);U\right)\right]}^{T}\right\}\;\\ & =\sum_{i=1}^{N_{T}}\sum_{j=1}^{N_{R}}\frac{8\pi^{2}\sigma^{4}}{\sigma_{n}^{2}\left(\sigma^{2}+\sigma_{n}^{2}\right)}\left[1+2h_{ij}+\frac{2h_{ij}}{\left(\sigma^{2}/\sigma_{n}^{2}\right)}\right]\times \left[\begin{array}{cc} \varepsilon_{i} & \gamma_{ij}\\ \gamma_{ij} & \eta_{ij} \end{array}\right], \end{array}$$

where the terms $\varepsilon_{i}$ , $\eta_{ij}$ , and $\gamma_{ij}$ are dependent on the radar waveforms, which can be calculated as:

(23) $$\begin{array}{cc} \varepsilon_{i} & \equiv \int_{-\infty }^{+\infty }f^{2}{\left|S_{i}\left(f\right)\right|}^{2}df-{\left|\int_{-\infty }^{+\infty }f{\left|S_{i}\left(f\right)\right|}^{2}df\right|}^{2}\;\\ & =\frac{1}{3}\pi^{2}k^{2}T^{2}, \end{array}$$

(24) $$\begin{array}{cc} \eta_{ij} & \equiv \int_{-\infty }^{+\infty }t^{2}{\left|s_{i}\left(t\right)\right|}^{2}df-{\left|\int_{-\infty }^{+\infty }t{\left|s_{i}\left(t\right)\right|}^{2}df\right|}^{2}\;\\ & =\frac{1}{12}T^{2}+\frac{1}{12}T_{R}^{2}\left(N^{2}-1\right), \end{array}$$

(25) $$\begin{array}{cc} \gamma_{ij} & \equiv ℑ\left\{\int_{-\infty }^{+\infty }ts_{i}^{*}\left(t\right)\frac{\partial s_{i}\left(t\right)}{\partial t}dt\right\}-\int_{-\infty }^{+\infty }t{\left|s_{i}\left(t\right)\right|}^{2}dt\int_{-\infty }^{+\infty }s_{i}\left(t\right)\frac{\partial s_{i}^{*}\left(t\right)}{\partial t}dt\;\\ & =-\frac{1}{6}k\pi T^{2}. \end{array}$$

The derivation of J(Q) is provided in Appendix A. Then, we can write the final expression for total FIM across all the transmitter-receiver pairs as:

(26) $$J\left(U\right)=\sum_{i=1}^{N_{T}}\sum_{j=1}^{N_{R}}\frac{8\pi^{2}\sigma^{4}}{\sigma_{n}^{2}\left(\sigma^{2}+\sigma_{n}^{2}\right)}\left[1+2h_{ij}+\frac{2h_{ij}}{\left(\sigma^{2}/\sigma_{n}^{2}\right)}\right]J_{ij}\left(U\right).$$

The expressions for the elements of the bistatic FIM $J_{ij}\left(U\right)$ corresponding to the ij th transceiver pair are given in Appendix B. The CRLB for the joint position and velocity estimation of a Rician target can be obtained by taking inverse of FIM in (26), i.e.,

(27) $$\begin{array}{c} \mathrm{CRLB}\left(U\right)=J^{-1}\left(U\right). \end{array}$$

**Remark** **1.** *It is obvious that the final expression of FIM in (26) is a linear combination of FIMs due to DS component and WIS components [23]. In this paper, one of our goals is to increase the SNR value at the radar receiver by employing the DS component, which leads to lower radar transmit power and better target estimation performance.*

**Remark** **2.** *From (26) and (27), we can observe that the MCRLB depends on a number of factors. It not only depends on the relative geometry between the target and the radar networks, but also depends on the transmitted LFM waveform parameters such as the duration of each pulse and bandwidth. In addition, it shows dependence on the target’s RCS and the SNR.*

## 4. Simulation Results

In the following, numerical results are dedicated to compute the joint CRLB for active active radar networks as well as reveal the effects of several factors on the CRLB.

**Figure 1:**
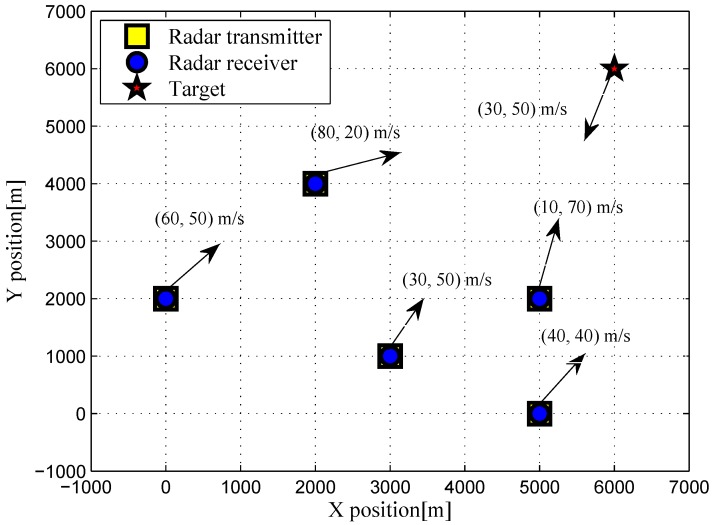
Target and radar networks configuration used in the numerical simulations.

For numerical simulations, we consider a radar network with five active radar transmitters and an equal number of multichannel receivers, i.e., $N_{T}=5$ and $N_{R}=5$ . The Cartesian coordinates of their positions are provided in Figure 1. The position/moving parameters of the radar transmitters are shown in Table 1. The receivers are co-located with the corresponding transmitters and have the same velocities. It is assumed that the target is located at [6000,6000] m with velocity [30,50] m/s. For simulation parameters, we set the LFM signal parameters as follows [29]: the number of subpulses N=256 , the bandwidth B=50 MHz, the duration of each pulse T=1 μs , the PRI $T_{R}=0.1$ ms, and the carrier wavelength λ=0.03 m.

Define the SNR as:

(28) $$\mathrm{SNR}=10\log\left(\frac{\sigma^{2}}{\sigma_{n}^{2}}\right).$$

**Figure 2:**
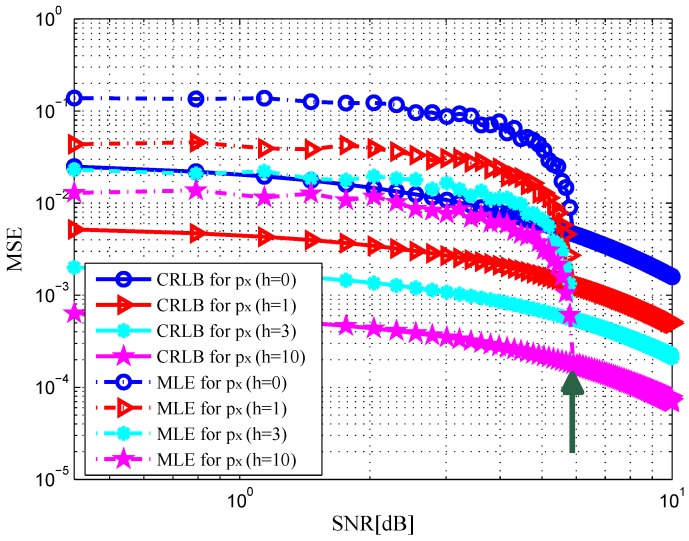
MSE versus SNR for x-dimension of target position with different *h*.

**Figure 3:**
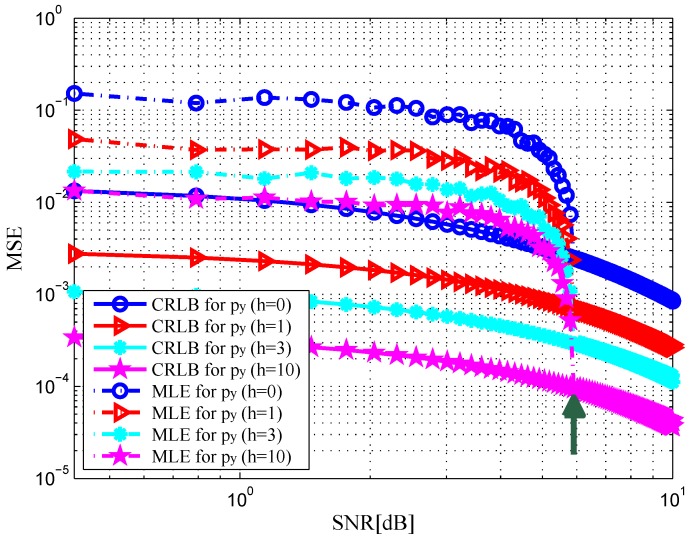
MSE versus SNR for y-dimension of target position with different *h*.

**Figure 4:**
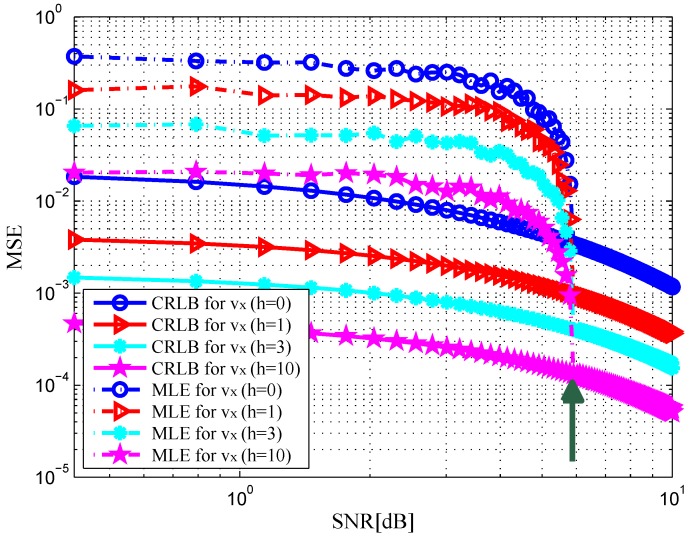
MSE versus SNR for x-dimension of target velocity with different *h*.

**Figure 5:**
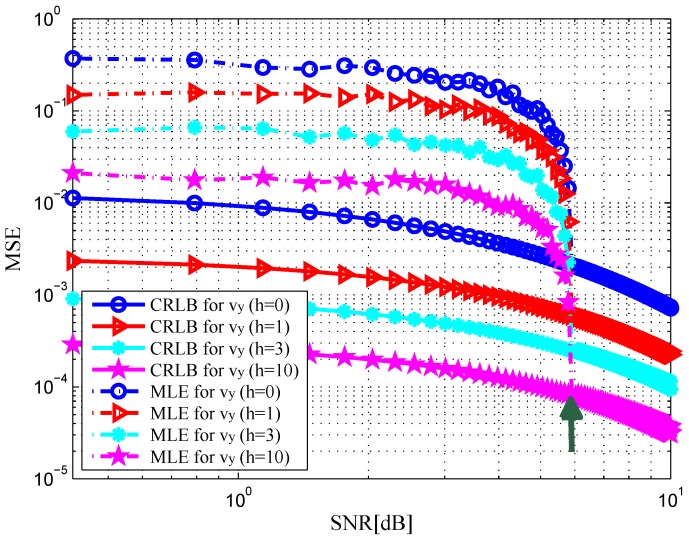
MSE versus SNR for y-dimension of target velocity with different *h*.

Without loss of generality, we assume that the reflection coefficients are the same for all transmitter-receiver pairs, i.e., $h_{ij}=h$ . In Figure 2 and Figure 3, the MSE curves are plotted versus SNR in the x-dimension and y-dimension of target position with different *h*. Solid and dashed curves show the CRLBs and the MSE curves of the ML estimation, respectively. As indicated in [12], it can be observed that the MSE is close to the CRLB in value and slope at an SNR threshold, see the green arrows in the figures. Similarly, we depict the MSE curves of target velocity against SNR in Figure 4 and Figure 5. From Figure 2, Figure 3, Figure 4 and Figure 5, we can notice that as the value of SNR goes up, the MSE decreases for both target position and velocity estimates.

In addition, it should be pointed out that the CRLB decreases significantly with an increase in *h*. This is due to the fact that an increase in *h* provides a rise in target RCS [23], which leads to the increase in the received SNR at the radar receiver. The CRLB will achieve a maximum value when DS component does not exist, i.e., h=0 , where the target RCS follows Rayleigh fluctuations in a non-coherent mode for all the transmitter-receiver pairs. In contrast, the CRLB will be minimum at an asymptotic limit, i.e., h→∞ , and the target is idealistically a point target in a coherent mode, which has a fixed amplitude RCS value for all the transmitter-receiver pairs. For the rest of the other cases, the CRLB lies in between these two values [23].

**Figure 6:**
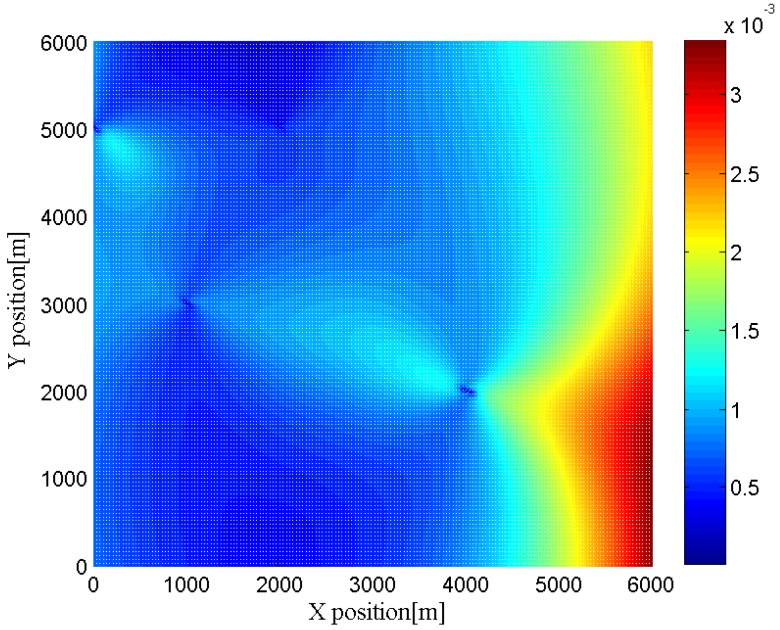
CRLB for x-dimension of target position in different position when SNR = 0 dB, h=3 .

**Figure 7:**
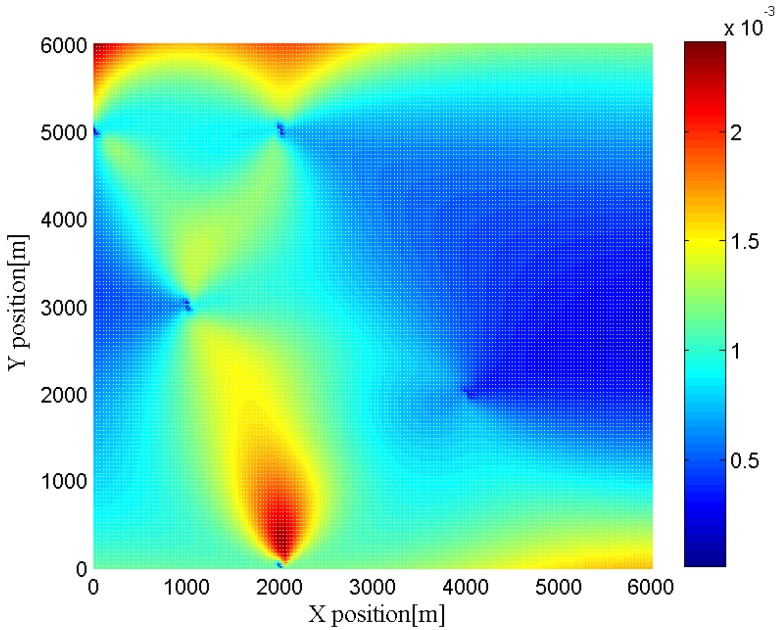
CRLB for y-dimension of target position in different position when SNR = 0 dB, h=3 .

**Figure 8:**
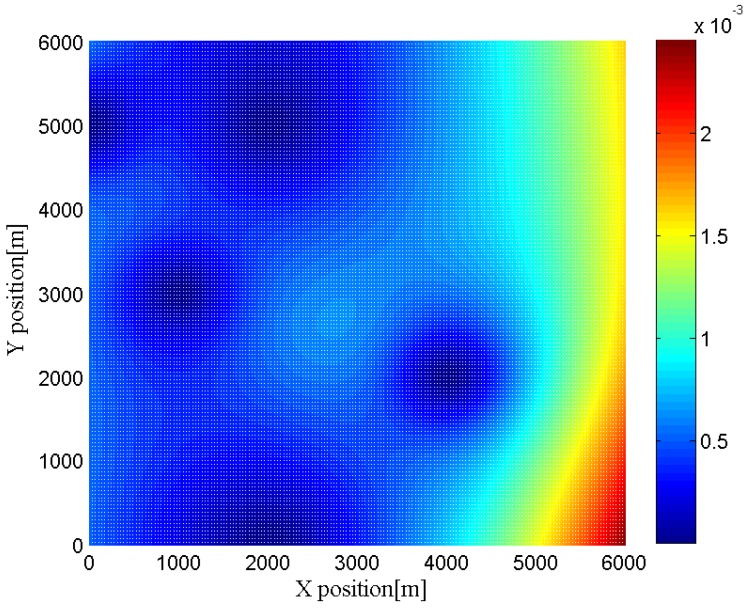
CRLB for x-dimension of target velocity in different position when SNR = 0 dB, h=3 .

**Figure 9:**
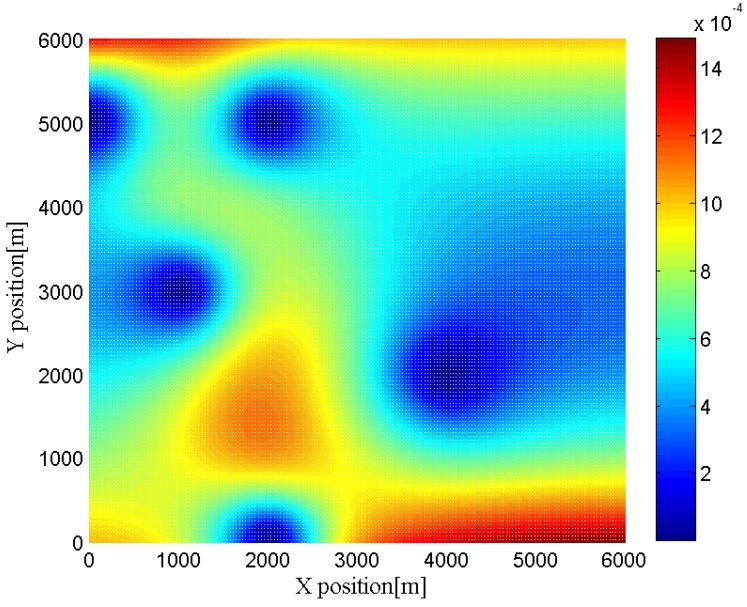
CRLB for y-dimension of target velocity in different position when SNR = 0 dB, h=3 .

Furthermore, we change the location of the target to different positions to investigate the effects of the geometry between the target and the radar networks. In Figure 6, Figure 7, Figure 8 and Figure 9, we show the CRLBs for both target position and velocity in different position when SNR = 0 dB, h=3 . From these results, we can observe that the CRLBs on the Cartesian coordinates of target position and velocity are different when the target is in different positions. This is because the geometry between the target and the radar network systems impacts the derivatives of the delay-Doppler terms with respect to the Cartesian coordinates significantly [20,23].

**Figure 10:**
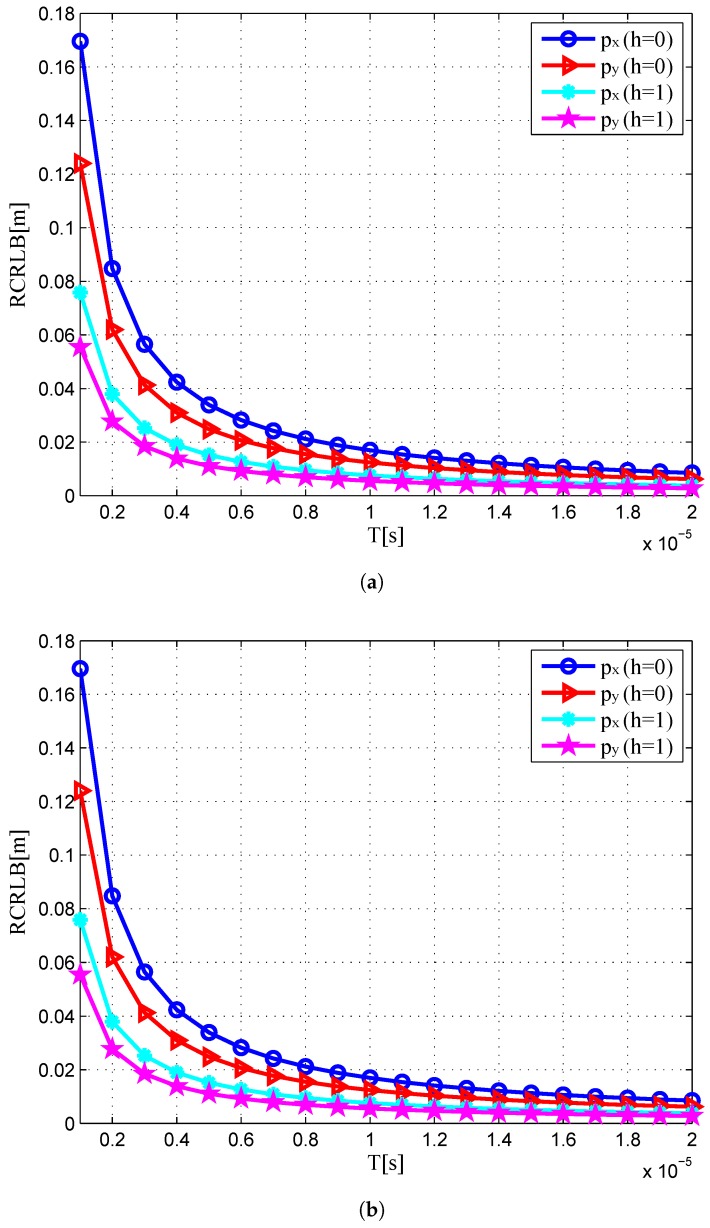
RCRLB in the target position dimensions versus waveform parameters when SNR = 0 dB with different *h*: (**a**) *T*; (**b**) *B*.

In Figure 10, we depict the square root of CRLBs (RCRLBs) for target position coordinates against the duration time of each pulse *T* and bandwidth *B* at 0 dB with different *h*. One can notice that the RCRLBs reduce as the waveform parameters increase, confirming that a waveform with a larger time-bandwidth product can provide better target estimation performance. It is worth mentioning that the figures of target velocity are omitted for the sake of brevity, which are similar to the figures of target position. Overall, it can be concluded that the CRLB shows dependence on the SNR, target’s RCS, geometry and waveform parameters.

## 5. Conclusions

In this paper, we examined the problem of moving target parameter estimation for active radar network systems with sensors on moving platforms in a Rice fading environment, which consist of multiple radar transmitters and multichannel receivers. The CRLB for joint position and velocity estimation of a Rician target has been derived. It should be noted that the cumulative FIM is a linear combination of both DS component and WIS components. Numerical examples have been provided to demonstrate that the joint target parameter estimation accuracy of active radar networks can be significantly improved with the exploitation of the DS component. Furthermore, it is shown that the joint CRLB is a function of the transmitted waveforms as well as the geometry between the target and the radar networks. Also, it depends on the SNR and target’s RCS. In future work, we will utilize this framework to investigate the problem of optimal power allocation of the radar networks in a Rice fading environment.

multiple

## Figures and Tables

**Table 1:** Location and Moving Parameters of the Radar Transmitters.

Transmitter Index	Locations [m]	Velocities [m/s]
Transmitter1	[3000,1000]	[30,50]
Transmitter2	[5000,2000]	[10,70]
Transmitter3	[2000,4000]	[80,20]
Transmitter4	[0,2000]	[60,50]
Transmitter5	[5000,0]	[40,40]

## Appendix A. The Derivation of FIM J(Q)

We have:

(A1)
$$\Gamma_{ij}^{1}=\frac{\sigma^{2}}{\sigma^{2}+\sigma_{n}^{2}}{\left|\int_{-\infty }^{+\infty }r_{ij}\left(t\right)s_{i}^{*}\left(t-\tau_{ij}\right)e^{-j2\pi f_{D_{ij}}\left(t-\tau_{ij}\right)}dt\right|}^{2}.$$

Let us suppose $\xi_{ij}=\int_{-\infty }^{+\infty }r_{ij}\left(t\right)s_{i}^{*}\left(t-\tau_{ij}\right)e^{-j2\pi f_{D_{ij}}\left(t-\tau_{ij}\right)}dt$ , then the first derivative with respect to $\tau_{ij}$ is given by:

(A2)
$$\begin{array}{cc} \frac{\partial \Gamma_{ij}^{1}}{\partial \tau_{ij}} & =\frac{\sigma^{2}}{\sigma^{2}+\sigma_{n}^{2}}\left(\xi_{ij}^{*}\frac{\partial \xi_{ij}}{\partial \tau_{ij}}+\xi_{ij}\frac{\partial \xi_{ij}^{*}}{\partial \tau_{ij}}\right)\;\\ & =\frac{2\sigma^{2}}{\sigma_{n}^{2}\left(\sigma^{2}+\sigma_{n}^{2}\right)}\times ℜ\left(\xi_{ij}\int_{-\infty }^{+\infty }r_{ij}^{*}\left(t\right)\frac{\partial s_{i}\left(t-\tau_{ij}\right)}{\partial \tau_{ij}}e^{j2\pi f_{D_{ij}}\left(t-\tau_{ij}\right)}\right). \end{array}$$

To compute the expectation with respect to the second-order derivative, we have:

(A3)
$$\begin{array}{cc} -E\left(\frac{\partial^{2}\Gamma_{ij}^{1}}{\partial \tau_{ij}^{2}}\right) & =-\frac{2\sigma^{2}\left(\sigma^{2}+d_{ij}^{2}\right)}{\sigma^{2}+\sigma_{n}^{2}}\times E\left[ℜ\left({\left|\int_{-\infty }^{+\infty }s_{i}\left(t-\tau_{ij}\right)\frac{\partial s_{i}^{*}\left(t-\tau_{ij}\right)}{\partial \tau_{ij}}dt\right|}^{2}\right.\right.\;\\ & \left.\left.+\int_{-\infty }^{+\infty }{\left|s_{i}\left(t-\tau_{ij}\right)\right|}^{2}dt\int_{-\infty }^{+\infty }s_{i}^{*}\left(t-\tau_{ij}\right)\frac{\partial^{2}s_{i}\left(t-\tau_{ij}\right)}{\partial \tau_{ij}^{2}}dt\right)\right]. \end{array}$$

With the derivations in [29,30], hence we obtain:

(A4)
$$-E\left(\frac{\partial^{2}\Gamma_{ij}^{1}}{\partial \tau_{ij}^{2}}\right)=\frac{8\pi^{2}\sigma^{4}}{\sigma_{n}^{2}\left(\sigma^{2}+\sigma_{n}^{2}\right)}\left(1+2h_{ij}\right)\varepsilon_{i},$$

where $h_{ij}={\left|d_{ij}\right|}^{2}/\left(2\sigma^{2}\right)$ .

To calculate the expectation with respect to other second-order derivatives, we follow the same procedure and arrive at the following closed form expression:

(A5)
$$-E\left(\frac{\partial^{2}\Gamma_{ij}^{1}}{\partial f_{D_{ij}}^{2}}\right)=\frac{8\pi^{2}\sigma^{4}}{\sigma_{n}^{2}\left(\sigma^{2}+\sigma_{n}^{2}\right)}\left(1+2h_{ij}\right)\eta_{ij}.$$

The off-diagonal terms can be obtained as:

(A6)
$$-E\left(\frac{\partial^{2}\Gamma_{ij}^{1}}{\partial \tau_{ij}\partial f_{D_{ij}}}\right)=\frac{8\pi^{2}\sigma^{4}}{\sigma_{n}^{2}\left(\sigma^{2}+\sigma_{n}^{2}\right)}\left(1+2h_{ij}\right)\gamma_{ij}.$$

Following the same lines, we can get the expectation of second-order derivatives of $\Gamma_{ij}^{2}$ and $\Gamma_{ij}^{3}$ as follows:

(A7)
$$-E\left(\frac{\partial^{2}\Gamma_{ij}^{2}}{\partial \tau_{ij}^{2}}\right)=\frac{8\pi^{2}\sigma^{4}}{\sigma_{n}^{2}\left(\sigma^{2}+\sigma_{n}^{2}\right)}\left(-\frac{2h_{ij}}{\sigma^{2}/\sigma_{n}^{2}}\right)\varepsilon_{i},$$

(A8)
$$-E\left(\frac{\partial^{2}\Gamma_{ij}^{2}}{\partial f_{D_{ij}}^{2}}\right)=\frac{8\pi^{2}\sigma^{4}}{\sigma_{n}^{2}\left(\sigma^{2}+\sigma_{n}^{2}\right)}\left(-\frac{2h_{ij}}{\sigma^{2}/\sigma_{n}^{2}}\right)\eta_{ij},$$

(A9)
$$-E\left(\frac{\partial^{2}\Gamma_{ij}^{2}}{\partial \tau_{ij}\partial f_{D_{ij}}}\right)=\frac{8\pi^{2}\sigma^{4}}{\sigma_{n}^{2}\left(\sigma^{2}+\sigma_{n}^{2}\right)}\left(-\frac{2h_{ij}}{\sigma^{2}/\sigma_{n}^{2}}\right)\gamma_{ij},$$

(A10)
$$-E\left(\frac{\partial^{2}\Gamma_{ij}^{3}}{\partial \tau_{ij}^{2}}\right)=\frac{8\pi^{2}\sigma^{4}}{\sigma_{n}^{2}\left(\sigma^{2}+\sigma_{n}^{2}\right)}\left(\frac{4h_{ij}}{\sigma^{2}/\sigma_{n}^{2}}\right)\varepsilon_{i},$$

(A11)
$$-E\left(\frac{\partial^{2}\Gamma_{ij}^{3}}{\partial f_{D_{ij}}^{2}}\right)=\frac{8\pi^{2}\sigma^{4}}{\sigma_{n}^{2}\left(\sigma^{2}+\sigma_{n}^{2}\right)}\left(\frac{4h_{ij}}{\sigma^{2}/\sigma_{n}^{2}}\right)\eta_{ij},$$

(A12)
$$-E\left(\frac{\partial^{2}\Gamma_{ij}^{3}}{\partial \tau_{ij}\partial f_{D_{ij}}}\right)=\frac{8\pi^{2}\sigma^{4}}{\sigma_{n}^{2}\left(\sigma^{2}+\sigma_{n}^{2}\right)}\left(\frac{4h_{ij}}{\sigma^{2}/\sigma_{n}^{2}}\right)\gamma_{ij}.$$

## Appendix B. The Elements of FIM J_*ij*_(U)

The elements of the symmetric FIM $J_{ij}\left(U\right)$ corresponding to the ij th transmitter-receiver pair are given by:

(B1)
$$J_{ij}^{11}\left(U\right)=\frac{k^{2}\pi^{2}T^{2}}{3}{\left(\frac{\partial \tau_{ij}}{\partial x}\right)}^{2}-\frac{k\pi T^{2}}{3}\left(\frac{\partial \tau_{ij}}{\partial x}\right)\left(\frac{\partial f_{D_{ij}}}{\partial x}\right)+\frac{1}{12}\left[T^{2}+T_{R}\left(N^{2}-1\right)\right]{\left(\frac{\partial f_{D_{ij}}}{\partial x}\right)}^{2},$$

(B2)
$$\begin{array}{cc} J_{ij}^{12}\left(U\right) & =J_{ij}^{21}\left(U\right)=\left\{\frac{k^{2}\pi^{2}T^{2}}{3}\left(\frac{\partial \tau_{ij}}{\partial x}\right)-\frac{k\pi T^{2}}{6}\left(\frac{\partial f_{D_{ij}}}{\partial x}\right)\right\}\left(\frac{\partial \tau_{ij}}{\partial y}\right)\;\\ & +\left\{-\frac{k\pi T^{2}}{6}\left(\frac{\partial \tau_{ij}}{\partial x}\right)+\frac{1}{12}\left[T^{2}+T_{R}\left(N^{2}-1\right)\right]\left(\frac{\partial f_{D_{ij}}}{\partial x}\right)\right\}\left(\frac{\partial f_{D_{ij}}}{\partial y}\right), \end{array}$$

(B3)
$$J_{ij}^{13}\left(U\right)=J_{ij}^{31}\left(U\right)=\left\{-\frac{k\pi T^{2}}{6}\left(\frac{\partial \tau_{ij}}{\partial x}\right)+\frac{1}{12}\left[T^{2}+T_{R}\left(N^{2}-1\right)\right]\left(\frac{\partial f_{D_{ij}}}{\partial x}\right)\right\}\left(\frac{\partial f_{D_{ij}}}{\partial v_{x}}\right),$$

(B4)
$$J_{ij}^{14}\left(U\right)=J_{ij}^{41}\left(U\right)=\left\{-\frac{k\pi T^{2}}{6}\left(\frac{\partial \tau_{ij}}{\partial x}\right)+\frac{1}{12}\left[T^{2}+T_{R}\left(N^{2}-1\right)\right]\left(\frac{\partial f_{D_{ij}}}{\partial x}\right)\right\}\left(\frac{\partial f_{D_{ij}}}{\partial v_{y}}\right),$$

(B5)
$$J_{ij}^{22}\left(U\right)=\frac{k^{2}\pi^{2}T^{2}}{3}{\left(\frac{\partial \tau_{ij}}{\partial y}\right)}^{2}-\frac{k\pi T^{2}}{3}\left(\frac{\partial \tau_{ij}}{\partial y}\right)\left(\frac{\partial f_{D_{ij}}}{\partial y}\right)+\frac{1}{12}\left[T^{2}+T_{R}\left(N^{2}-1\right)\right]{\left(\frac{\partial f_{D_{ij}}}{\partial y}\right)}^{2},$$

(B6)
$$J_{ij}^{23}\left(U\right)=J_{ij}^{32}\left(U\right)=\left\{-\frac{k\pi T^{2}}{6}\left(\frac{\partial \tau_{ij}}{\partial y}\right)+\frac{1}{12}\left[T^{2}+T_{R}\left(N^{2}-1\right)\right]\left(\frac{\partial f_{D_{ij}}}{\partial y}\right)\right\}\left(\frac{\partial f_{D_{ij}}}{\partial v_{x}}\right),$$

(B7)
$$J_{ij}^{24}\left(U\right)=J_{ij}^{42}\left(U\right)=\left\{-\frac{k\pi T^{2}}{6}\left(\frac{\partial \tau_{ij}}{\partial y}\right)+\frac{1}{12}\left[T^{2}+T_{R}\left(N^{2}-1\right)\right]\left(\frac{\partial f_{D_{ij}}}{\partial y}\right)\right\}\left(\frac{\partial f_{D_{ij}}}{\partial v_{y}}\right),$$

(B8)
$$J_{ij}^{33}\left(U\right)=\frac{1}{12}\left[T^{2}+T_{R}\left(N^{2}-1\right)\right]{\left(\frac{\partial f_{D_{ij}}}{\partial v_{x}}\right)}^{2},$$

(B9)
$$J_{ij}^{34}\left(U\right)=J_{ij}^{43}\left(U\right)=\frac{1}{12}\left[T^{2}+T_{R}\left(N^{2}-1\right)\right]\left(\frac{\partial f_{D_{ij}}}{\partial v_{x}}\right)\left(\frac{\partial f_{D_{ij}}}{\partial v_{y}}\right),$$

(B10)
$$J_{ij}^{44}\left(U\right)=\frac{1}{12}\left[T^{2}+T_{R}\left(N^{2}-1\right)\right]{\left(\frac{\partial f_{D_{ij}}}{\partial v_{y}}\right)}^{2}.$$
